# Clinical relevance of postzygotic mosaicism in Cornelia de Lange syndrome and purifying selection of *NIPBL* variants in blood

**DOI:** 10.1038/s41598-021-94958-z

**Published:** 2021-07-29

**Authors:** Ana Latorre-Pellicer, Marta Gil-Salvador, Ilaria Parenti, Cristina Lucia-Campos, Laura Trujillano, Iñigo Marcos-Alcalde, María Arnedo, Ángela Ascaso, Ariadna Ayerza-Casas, Rebeca Antoñanzas-Pérez, Cristina Gervasini, Maria Piccione, Milena Mariani, Axel Weber, Deniz Kanber, Alma Kuechler, Martin Munteanu, Katharina Khuller, Gloria Bueno-Lozano, Beatriz Puisac, Paulino Gómez-Puertas, Angelo Selicorni, Frank J. Kaiser, Feliciano J. Ramos, Juan Pié

**Affiliations:** 1 Unit of Clinical Genetics and Functional Genomics, Department of Pharmacology-Physiology, School of Medicine, Universidad de Zaragoza, CIBERER-GCV02 and IIS-Aragon, 50009 Zaragoza, Spain; 2 Institut für Humangenetik, Universitätsklinikum Essen, Universität Duisburg-Essen, Essen, Germany; 3 Unit of Clinical Genetics, Service of Paediatrics, Hospital Clínico Universitario Lozano Blesa, Department of Paediatrics, School of Medicine, Universidad de Zaragoza, CIBERER-GCV02 and IIS-Aragon, 50009 Zaragoza, Spain; 4 Molecular Modelling Group, Centro de Biología Molecular Severo Ochoa, CBMSO (CSIC-UAM), 28049 Madrid, Spain; 5 Biosciences Research Institute, School of Experimental Sciences, Universidad Francisco de Vitoria, 28223 Pozuelo de Alarcón, Madrid, Spain; 6 Unit of Paediatric Cardiology, Service of Paediatrics, Hospital Universitario Miguel Servet, 50009 Zaragoza, Spain; 7 Genetica Medica, Dipartimento di Scienze della Salute, Università degli Studi di Milano, Milano, Italy; 8 Department of Health Promotion, Mother and Child Care, Internal Medicine and Medical Specialties, University of Palermo, Palermo, Italy; 9 Centro Fondazione Mariani per il Bambino Fragile, Department of Pediatrics, ASST-Lariana Sant’Anna Hospital, San Fermo della Battaglia (Como), Italy; 10 Institute of Human Genetics, Justus-Liebig-University, Giessen, Germany; 11 Essener Zentrum für Seltene Erkrankungen (EZSE), Universitätsmedizin Essen, Universitätsklinikum Essen, Essen, Germany

**Keywords:** Diseases, Medical research, Genetics research, Paediatric research

## Abstract

Postzygotic mosaicism (PZM) in *NIPBL* is a strong source of causality for Cornelia de Lange syndrome (CdLS) that can have major clinical implications. Here, we further delineate the role of somatic mosaicism in CdLS by describing a series of 11 unreported patients with mosaic disease-causing variants in *NIPBL* and performing a retrospective cohort study from a Spanish CdLS diagnostic center. By reviewing the literature and combining our findings with previously published data, we demonstrate a negative selection against somatic deleterious *NIPBL* variants in blood. Furthermore, the analysis of all reported cases indicates an unusual high prevalence of mosaicism in CdLS, occurring in 13.1% of patients with a positive molecular diagnosis. It is worth noting that most of the affected individuals with mosaicism have a clinical phenotype at least as severe as those with constitutive pathogenic variants. However, the type of genetic change does not vary between germline and somatic events and, even in the presence of mosaicism, missense substitutions are located preferentially within the HEAT repeat domain of NIPBL. In conclusion, the high prevalence of mosaicism in CdLS as well as the disparity in tissue distribution provide a novel orientation for the clinical management and genetic counselling of families.

## Introduction

Genetic mosaicism is a well-described biological phenomenon characterized by the presence of genetically distinct lineages of cells in the same individual due to postzygotic de novo mutational events. Far from being an exceptional condition, technical advances in DNA and RNA sequencing, which can even sequence a single cell, have confirmed the theoretical hypothesis that mosaicism is the norm in humans^1–3^. Postzygotic mosaicism (PZM) can refer to a variety of different mutation types, such as single-nucleotide substitutions, insertions, deletions, and copy-number variants (CNVs). The biological consequences of these mutations are mainly determined by their developmental timing, as well as the type and fraction of the affected tissue. Thus, mosaic mutations can go unnoticed, contribute to human variation, promote cancer, be involved in aging or underlie genetic diseases. In this context, several reviews have been published discussing the implication of mosaicism in human health and disease^4–8^.

It has long been known that genetic mosaicism appears in a wide range of clinical disorders. Due to the technical challenges inherent detecting mosaicism, the first cases were described in the 60s in patients with chromosomal disorders, such as in Klinefelter and Turner syndromes^9,10^. As the sensitivity of detection of PZM has been increasing over the last few years, many new cases of well-known monogenic disorders caused by mosaic variants have been described. For Cornelia de Lange syndrome (CdLS), whose first case of PZM was described in 2010^11^, more than 30 cases have been reported so far^12,13^.

CdLS (OMIM #122470, #300590, #610759, #614701, #300882) is a rare, congenital disorder characterized by a widely variable clinical presentation. The majority of affected individuals present prenatal and postnatal growth retardation, characteristic facial dysmorphic features, intellectual disability and limb reduction defects^14^. Around 60–70% of affected individuals harbor a heterozygous loss-of-function pathogenic variant in the cohesin loading factor *NIPBL*, and approximately 5–10% of the cases have been associated with seven additional genes related to the cohesin complex (*SMC1A, SMC3, RAD21*, *HDAC8, BRD4, ANKRD11* and *MAU2*)^15–20^. Moreover, pathogenic variants in other key chromatin-associated factors, such as *ARID1B*, *SMARCB1*, *EP300* or *KMT2A*, have been described in patients presenting features of CdLS or CdLS-like phenotypes^21–23^. Strikingly, mosaic pathogenic variants are found in *NIPBL* in many of the undiagnosed individuals in the first analysis^24^. Additionally, in contrast to what is commonly seen in other conditions, individuals with mosaic variants present with clinical features that are as severe as those observed in individuals harbouring constitutive pathogenic variants, and the two groups are clinically indistinguishable^13,25^.

The relevance of PZM in the pathogenesis of CdLS and its contribution to the phenotypic presentation remain largely unexplored. Moreover, the prevalence of PZM in CdLS also requires further examination through large-scale studies. The accurate and comprehensive categorization and subtyping of CdLS based on heritability has relevant clinical implications for genetic counselling of families. Therefore, the main aim of this study is to discuss and expand on the crucial role of genetic mosaicism in CdLS. Here, we present 11 patients with mosaic disease-causing variants in *NIPBL*, and compare the data of our cohort with those available in the literature to perform a robust and detailed evaluation of the mosaicism status in CdLS. In addition, we have explored the prevalence of PZM in CdLS in a retrospective study of a cohort of patients diagnosed in our reference centre for CdLS in Spain.

## Results

### Novel postzygotic mosaic variants in 11 individuals with Cornelia de Lange syndrome

Here we report a total of 11 new cases of postzygotic mosaicism in individuals with CdLS from Germany, Italy and Spain. Based on their clinical CdLS score, 10 individuals showed classic CdLS phenotypes and only one showed a non-classic phenotype (Table 1). Patients reported here had consistent global developmental delay and intellectual disability (10/11). All of them presented the characteristic (classic) CdLS craniofacial features such as synophrys, thick arched eyebrows, thin upper lip vermilion and downturned corners of mouth (11/11). Upturned nasal tip (9/11) and elongated smooth *philtrum* (10/11) were also commonly observed. Regarding growth parameters, microcephaly (9/11) and postnatal growth retardation (9/11) were the anomalies most frequently observed (Table 1). All 11 individuals presented mosaic disease-causing variants in *NIPBL*. Using next generation sequencing (NGS), a total of seven novel and four previously reported *NIPBL* variants were detected. Alternative allele frequency (AAF) values ranged from 19 to 46.5% in buccal swab or skin fibroblasts DNA. Only in one individual the pathogenic variant could be detected in blood with an AAF higher than 2% (Table 2).

**Table 1:** Clinical findings of the 11 reported individuals with *NIPBL* mosaic variants.

#I	#I1	#I2	#I3	#I4	#I5	#I6	#I7	#I8	#I9	#I10	#I11
Sex/age	M/26	F/3	M/52	M/4	M/12	M/49	M/19	F/17	M/18	M/5	M/5
Origin	G	G	G	G	IT	IT	IT	IT	S	S	S
Clinical score	8	14	12	12	13	12	14	14	14	14	14
Synophrys (HP:0000664) and/or thick eyebrows (HP:0000574)	+	+	+	+	+	+	+	+	+	+	+
Short nose (HP:0003196), concave nasal ridge (HP:0011120) and/or upturned nasal tip(HP:0000463)	−	+	+	+	+	−	+	+	+	+	+
Long (HP:0000343) and/or smooth philtrum (HP:0000319)	−	+	+	+	+	+	+	+	+	+	+
Thin upper lip vermilion (HP:0000219) and/or downturned corners of mouth (HP:0002714)	+	+	+	+	+	+	+	+	+	+	+
Hand oligodactyly (HP:0001180) and/or adactyly (HP:0009776)	−	+	−	−	−	−	−	−	−	−	−
Congenital diaphragmatic hernia (HP:0000776)	−	−	−	−	−	−	−	−	−	−	−
Global developmental delay (HP:0001263) and/or intellectual disability (HP:0001249)	+	+	+	+	+	+	−	+	+	+	+
Prenatal growth retardation (< 2 sD) (HP:0001511)	+	−	−	+	−	+	+	−	−	+	−
Postnatal growth retardation (< 2 sD) (HP:0008897)	+	+	−	+	+	−	+	+	+	+	+
Microcephaly (prenatally and/or postnatally) (HP:0000252)	+	+	−	+	+	+	−	+	+	+	+
Small hands (HP:0200055) and/or feet (HP:0001773)	−	+	+	−	+	+	+	+	+	+	+
Short fifth finger (HP:0009237)	−	−	+	−	+	+	+	+	+	+	+
Hirsutism (HP:0001007)	−	−	+	−	−	+	+	+	+	−	+

*Abbreviations*: I, Individual; M, male; F, female; +, positive; −, negative; G, Germany; IT, Italy; S, Spain.

**Table 2:** Molecular findings of the 11 reported individuals with *NIPBL* mosaic variants.

#I	Gene	Genomic position (hg19)	DNA variation	Protein variation	Variation Type	Exon	Detection Method	AAF Blood	AAF Buccal Cells	AAF Fibroblasts	AAF Skeletal Muscle	Novel or Reported
#I1	*NIPBL*	chr5:36985576	c.2294G > A	p.(Arg765Lys)	missense	10	NGS Panel	26% (1232 reads)	35% SNaPshot	n.d	n.d	Novel
#I2	*NIPBL*	chr5:37048649	c.6635T > A	p.(Val2212Glu)	missense	39	NGS Panel	2% (2347 reads)	28% (294 reads)	n.d	n.d	Reported
#I3	*NIPBL*	chr5:37057352	c.7328_7329insA	p.(Glu2444Glyfs*19)	frameshift	43	WES	n.d	n.d	13% (187 reads)	n.d	Novel
#I4	*NIPBL*	chr5:37059203	c.7621delC	p.(Gln2541Argfs*9)	frameshift	44	WES	< 2% (159 reads)	26% (170 reads)	n.d	n.d	Novel
#I5	*NIPBL*	chr5:36953840	c.42delG	p.(Ile16Leufs*8)	frameshift	2	NGS Panel	n.d	21% (176 reads)	n.d	n.d	Novel
#I6	*NIPBL*	chr5:36972091_36972092	c.816_817delGA	p.(Arg273Ilefs*12)	frameshift	8	NGS Panel	n.d	24% (167 reads)	n.d	n.d	Novel
#I7	*NIPBL*	chr5:36955642	c.133C > T	p.(Arg45*)	nonsense	3	NGS Panel	n.d	19% (27 reads)	n.d	n.d	Reported
#I8	*NIPBL*	chr5:36985884	c.2602C > T	p.(Arg868*)	nonsense	10	NGS Panel	n.d	23% (26 reads)	n.d	n.d	Reported
#I9	*NIPBL*	chr5:37052573	c.7168G > A	p.(Ala2390Thr)	missense	42	NGS Panel	< 2% (1730 reads)	n.d	23% (1805 reads)	37.9% (2000 reads)	Reported
#I10	*NIPBL*	chr5:36986303	c.3021delA	p.(Lys1007Asnfs*37)	frameshift	10	NGS Panel	< 2% (4386 reads)	n.d	46.5% (1995 reads)	n.d	Novel
#I11	*NIPBL*	chr5:36985717	c.2435_2436insA	p.(Ser813Valfs*5)	frameshift	10	NGS Panel	< 2% (1995 reads)	n.d	35.9% (1999 reads)	n.d	Novel

NIPBL RefSeq NM_133433. Total number of reads is indicated. *Abbreviations*: I, Individual; AAF, alternative allele frequency; n.d., not determined.

### Review of published cases of postzygotic mosaicism in Cornelia de Lange syndrome

We surveyed the literature to look for all pathogenic PZM variants in CdLS reported at the time of preparation of this manuscript. Including the 11 patients described in this study, a total of 43 individuals with CdLS somatic mosaicism were identified and classified according to the affected gene: 38 *NIPBL*, 2 *SMC1A*, 1 *SMC3*, 1 *ANKRD11* and 1 *KMT2A* (Supplementary Table 1). For 26 of those 43 patients, clinical data were available. Twenty-two of them presented classic CdLS and four mild or non-classic CdLS. Fourteen out of the 43 mosaic variants found had been previously identified in heterozygosis in patients with CdLS (Supplementary Table 1).

### Characteristics of mosaic variants in *NIPBL*

**Figure 1:**
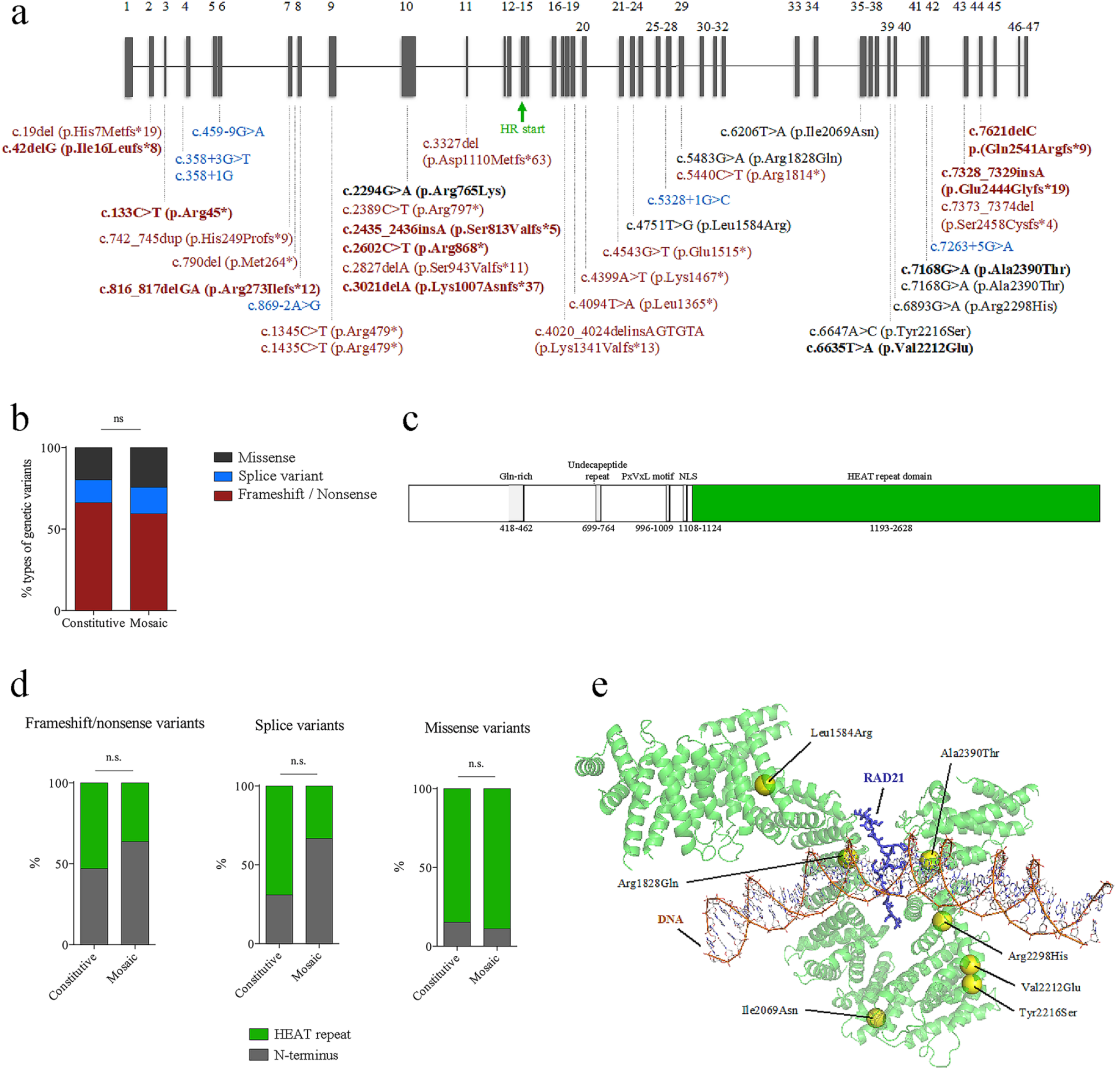
PZM in the *NIPBL* gene. (**a**) Schematic representation of the *NIPBL* gene including the localization of PZM variants described in the literature and the newly identified variants (in bold). Nonsense and frameshift variants are shown in red, splice variants in blue, and missense variants in black. Green arrow indicates the starting exon of the HEAT repeat domain (HR). (**b**) Proportion of nonsense/frameshift, splice and missense variants in ClinVar (constitutive, n = 299) and in the mosaic datasets (mosaic, n = 37). (**c**) Domain structure of NIPBL protein. HR domain is shown in green. (**d**) Distribution pattern of constitutive and mosaic variants in N-Terminal motif and HR of NIPBL according to genetic variant type, frameshift/nonsense (constitutive n = 198, mosaic n = 22), splice variants (constitutive n = 42, mosaic n = 6) or missense (constitutive n = 59, mosaic n = 9). (n.s. *p* > 0.05, **p < 0.01, ***p < 0.001, Chi-square test). (**e**) 3D representation of NIPBL HR (green) in close contact with a small segment of RAD21 (blue) and a DNA molecule, as described in the Protein Data Bank structure id: 6WGE. Position of variants Leu1584Arg, Arg1828Gln, Ile2069Asn, Val2212Glu, Tyr2216Ser, Arg2298His and Ala2390Thr is indicated (yellow spheres).

Postzygotic mosaic *NIPBL* variants are scattered across the entire gene. Only one variant was shared by two unrelated individuals: *NIPBL*, (RefSeq NM_133433), c.7168G > A; p.(Ala2390Thr) (Fig. 1a). One gross gene rearrangement causing a deletion of exons 2 to 32 was reported. Of the 37 point variants identified so far, 59.5% (22/37) are nonsense or frameshift variants, 16.2% (6/37) are splice variants, and 24.3% (9/37) are missense variants. A similar proportion of each type of variants was observed for de novo mutations (DNM) or germline variants found in *NIPBL* currently deposited in ClinVar (Fig. 1b). Interestingly eight of the nine mosaic missense substitutions are located within the HEAT repeat domain of *NIPBL* (Fig. 1c–e). A similar trend was observed for all the pathogenic and likely pathogenic constitutive variants described in ClinVar. It is noteworthy that nonsense, frameshift and splice variants are distributed all over the gene, while the vast majority of the missense variants are located in the HEAT repeat domain (Fig. 1c,d).

### Purifying selection against *NIPBL* disease-causing variants in blood

**Figure 2:**
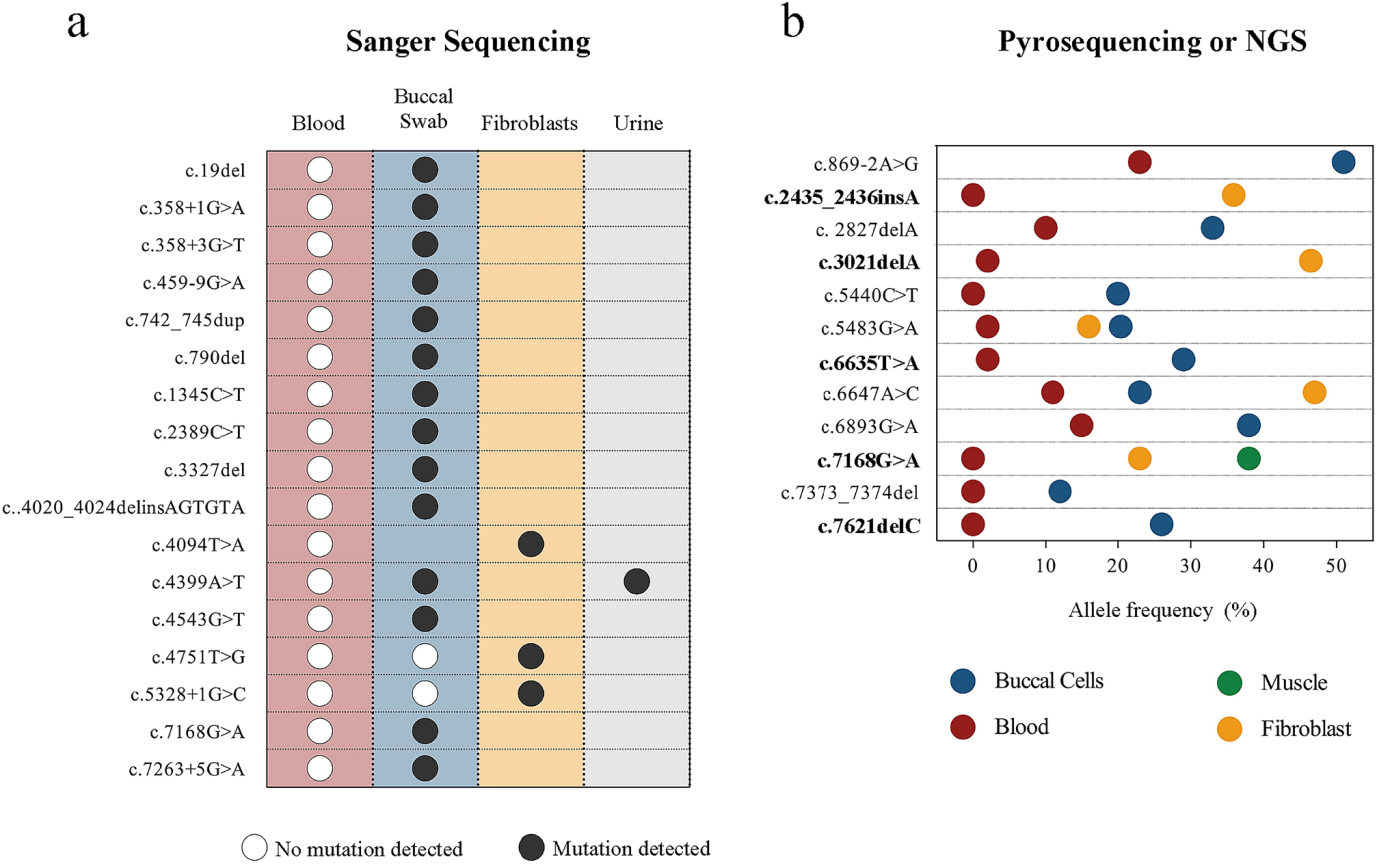
Summary of PZM sequencing results in different tissues: Picture illustrate individuals with at least two tissues analyzed. (**a**) Sanger sequencing results in 17 published patients with PZM variants in *NIPBL*. Dots indicate genetic variant detected (black) or not detected (white) in the different tissues analyzed. (**b**) The graph shows the alternative allele frequency of the PZM variants calculated by NGS or pyrosequencing in 12 patients (7 already published and 5 novel patients). Each line corresponds to a patient, colored dots indicate the tissue sample analyzed: blood (red), buccal swab (blue), fibroblasts (yellow) or skeletal muscle (green).

Mosaic variants were detected by Sanger sequencing, pyrosequencing and/or NGS on genomic DNA. Blood and at least one additional tissue (cultured skin fibroblasts, saliva and/or buccal swabs, urine or muscle) were analyzed in 29 out of the 38 cases with *NIPBL* mosaic variants. The detection of these 29 variants was achieved by quantitative methods (NGS and/or pyrosequencing) in 12 cases and by non-quantitative Sanger sequencing in 17 cases. For all 29 cases, the genetic change on blood DNA was present at a very low allelic frequency or was undetected (Fig. 2, Supplementary Table 1).

### High frequency of postzygotic mosaicism in Cornelia de Lange syndrome

**Figure 3:**
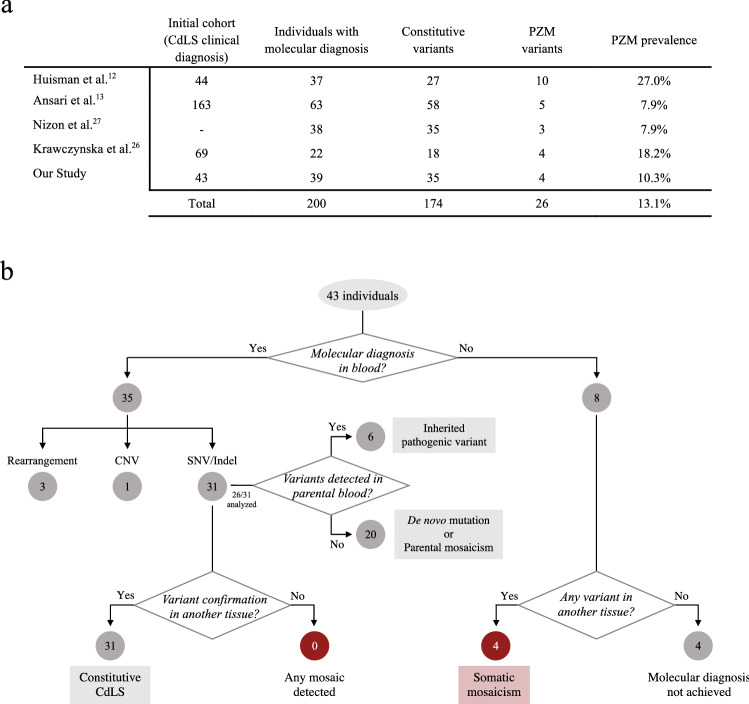
Prevalence of mosaicism in CdLS: (**a**) Percentage of patients with PZM among CdLS patients molecularly diagnosed in different studies. (**b**) Flow chart of genomic analysis in a cohort of 43 Spanish patients with clinical diagnosis of CdLS. The biological sample analyzed, the technique used for detecting variants or rearrangements, and the number of subjects carrying a pathogenic variant is indicated.

Out of the 12 studies identified in the literature on PZM in CdLS, four were cohort studies^12,13,26,27^. Due to differences in inclusion criteria among studies, we calculated the prevalence of PZM by dividing the reported number of patients with PZM by the total number of CdLS patients who received a molecular diagnosis. Across the studies, the frequency of PZM ranges from 7.9 to 27% (Fig. 3a). In order to accomplish a more comprehensive evaluation of the relevance of mosaicism in CdLS, we performed a detailed retrospective study in a Spanish cohort clinically diagnosed as CdLS. Of the 43 patients included, 39 were molecularly diagnosed (90.7%, 39/43). By Sanger sequencing and/or NGS targeted panel on DNA from blood, heterozygous causative variants in cohesin-related genes were found in 31 patients (72.1%, 31/43; of which 22/31 in *NIPBL*; 4/31 in *SMC1A*; 2/31 in *HDAC8*; 1/31 in *SMC3*; 1/31 in *RAD21*; and 1/31 in *ANKRD11*)^28–32^. The 31 causative variants identified in blood were confirmed in all patients by Sanger sequencing in at least one additional biological sample (saliva, buccal swabs or fibroblasts), confirming that the variants identified were all constitutive. By array CGH and MLPA, the genetic cause of the disorder was detected in four patients. Two of them presented a microdeletion involving the *RAD21*^33^ and *ARID1B*^23^ genes, respectively. One patient showed a duplication including the *SMC1A* gene^34^; and the fourth patient harbored a deletion of *NIPBL* exon 4. For the remaining eight undiagnosed patients, we applied targeted NGS panel on fibroblasts or saliva samples. A causative variant in *NIPBL* gene was detected in four of them. Notably none of the variants could be detected by Sanger sequencing on DNA derived from peripheral blood. At the end of our study, a molecular diagnosis could not be assigned in four cases (9.3%, 4/43). Hence the prevalence of somatic mosaicism in our cohort was 10.26% (4/39), when considering the individuals with a defined molecular diagnosis (Fig. 3b).

## Discussion

Currently, a molecular diagnosis is established in approximately 85%^14^ of patients with a clinical diagnosis of CdLS. An invaluable tool to reach this high percentage of solved cases is sensitive next generation sequencing, and in particular the incorporation of deep-sequencing target panels. By this, a set of genes can be analyzed simultaneously with very high sequencing depth, allowing the identification of genetic mosaicism, which is of special relevance in the context of CdLS^12,35,36^. Recently, it has been estimated that about 3% of causative de novo point variants in children with developmental disorders occurred as PZM^37^. So far, including the present work, five cohort studies have analyzed the prevalence of mosaicism in CdLS^12,13,26,27^. Across the studies, the frequency of PZM ranges from 7.9 to 27%. This variance could be explained by differences in the clinical characteristics of the patient cohort, the inclusion criteria, the molecular analyses performed and the tissues analyzed. Despite these limitations and the more than probable selection bias included in the retrospective studies, taking into account all five studies, PZM has been identified in 13.1% of the individuals who received a molecular diagnosis, which entails an unusual high frequency of somatic mosaicism in this genetic disease.

In other syndromes, many mosaic cases could go unnoticed inasmuch as, potentially, a mosaic variant causes a less severe and/or variable phenotype compared with the equivalent constitutive variant^4^. However, this is not the case for CdLS, since CdLS patients with somatic mosaicism may present with clinical manifestations as severe as individuals harboring a heterozygous loss-of-function variant in a known causative gene. In fact, 22 out of the 26 mosaic patients for whom clinical data were available (included the 11 reported in this paper), showed a classic CdLS phenotype.

In the vast majority of mosaic cases described in association with CdLS, *NIPBL* is the affected gene. The genetic variant type (frameshift, nonsense, missense or splice variant), as well as its distribution over the gene, do not appear to be influenced by the mosaic condition. However, it seems remarkable that the majority of missense variants found in *NIPBL* lie within the HEAT repeat domain, a very important region for the functionality of the protein. The structure of this domain was recently solved using cryo-electron microscopy (cryo-EM)^38^. It suggests an involvement in binding of a segment of the central unstructured domain of RAD21 as well as to the DNA molecule, thus reinforcing the hypothesis that the HEAT repeat domain plays a central role for the function of NIPBL and the cohesin complex. Somatic mosaicism was also reported for pathogenic variants in other CdLS-related genes^13,39–41^. Further studies based on deep sequencing are needed for a better characterization of mosaicism in non-*NIPBL* genes to withdraw conclusions about the frequency of mosaic variants in each CdLS causative gene. Besides CdLS, this phenomenon has been also described for other chromatinopathies, including Rubinstein–Taybi Syndrome (*CREBBP*)^42,43^, Wiedemann–Steiner Syndrome (*KMT2A*)^26^ or Coffin–Siris Syndrome (*ARID1A*)^44^.

Despite great progress in DNA sequencing techniques, mosaicism of pathogenic variants as cause of CdLS is frequently missed because genomic DNA from peripheral blood cells is used as the standard sample for routine genetic diagnostics. Unfortunately, the majority of mosaic events in CdLS were detected in DNA derived from buccal cells, saliva, urine, fibroblasts and/or skeletal muscle, whereas none of the cases described shows an overrepresentation of the mutant allele in DNA from peripheral blood. It could be thought that the explanation of this particularity relies on the fact that analyses of other tissues are only carried out when a pathogenic variant cannot be found in blood. Nevertheless, in this work we have analyzed other tissues in all the patients in whom causative variants had been detected in blood, but we did not identify any mosaic. That suggests PZM or genetic reversion followed by a negative selection against mutated clones in blood. Reversion is a rare phenomenon mainly described in skin and hematological diseases and associated with milder phenotypes than constitutive cases^45^. However, given the severity of mosaic cases in CdLS, the heterogeneous allele distribution observed amongst tissues and that back mutations would be unusually frequent for the various *NIPBL* causative variants, there are no evidences supporting this phenomenon in CdLS.

It is assumed that the extent of mosaicism across different tissues of a patient depends, at least in part, on the moment of occurrence of the mutation during early embryogenesis, the relative size of the founding population, and the cell fitness and quality. The clinical severity observed in mosaic CdLS patients suggests the arousal of the pathogenic variants early in development. More precisely, the presence of these variants in cells from different germ layers indicates that the mutational event might have taken place after zygotic stage but before gastrulation process. Thus, the specific absence of causative *NIPBL* variants in blood cannot be explained by the time of occurrence of the mutational event. Instead, it seems that the functional alterations in cells due to these variants could lie behind the mosaicism dynamics.

Several mechanisms of genetic selective pressures have been proposed. For example, DNA damage response or unfolded protein response are implicated in cell-autonomous elimination of altered cells, meanwhile innate immune system or local competitive interactions between neighboring cells may drive the expansion or elimination of cells harboring pathogenic variants in a cell non-autonomous manner^8^. Recently, it has been demonstrated that cells derived from CdLS patients display a defective DNA damage signaling and repair^46^. Actually, NIPBL is yet known to have important roles in 3D genome organization and stability^47^, and its knocking down has been directly correlated with higher levels of DNA damage^48^. It seems likely that mutated cell population could have a selective growth disadvantage over unaffected cells, leading to the expansion of the wild-type clones in bone marrow. A similar phenomenon of somatic rescue events specifically in blood has been demonstrated in some genome instability syndromes, such as Fanconi anemia or Bloom syndrome^49^, in which pathogenic variants in genes related to DNA damage and repair seem to reduce the fitness of hematopoietic stem and progenitor cells (HSPCs) and drive clonal selection and expansion of non-diseased cells^50^. By all means, a better understanding of mosaicism dynamics and the forces that drive the generation and shaping of somatic mosaicism in CdLS will provide new insights of a fundamental biological process and will enhance our understanding of the pathological mechanisms of this disease.

This phenomenon of negative selection against somatic deleterious variants in blood may be more common than reported so far. A recent massive RNA-seq analysis in samples from individuals of the Genotype-Tissue Expression (GTEx) cohort revealed that less than half of disease-causing mosaic variants in genes expressed in blood were detectable in blood-derived DNA^3^. Furthermore, selective genetic segregation in blood has been also described in some genetic disorders such as Pallister–Killian Syndrome^51^ or even in some mitochondrial diseases^52^, in which genetic testing begins from urine or fibroblasts samples instead of blood. The current recommendation for CdLS is to conduct a mosaicism study using fibroblasts, buccal cells or bladder epithelial cells when targeted panel or Sanger sequencing do not detect causal variants in lymphocytes^14,53^. We are well aware of the problems involved in collecting some biological samples and the technical challenges of obtaining high quality DNA from some of them. Thus, simultaneous collection of blood samples and buccal swabs may be a plausible option when a patient is suspected of having CdLS. Preferably, if the quality and quantity of DNA extracted from buccal swab sample meet the same standards as those established for blood samples, the first-line molecular testing should analyze DNA derived from buccal swab using a deep targeted gene panel containing at least the eight known CdLS causative genes. In case the panel detected a causal variant, this should be confirmed by Sanger sequencing in DNA derived from blood to evaluate mosaicism condition.

Besides the above considerations, the high prevalence of mosaicism as well as the disparity in tissue distribution can have major clinical implications in CdLS regarding parental counselling about recurrence risk. In principle, in routine clinical practice, the risk classification is made based on variant heritability: Hereditary (high risk), DNM (low risk) and PZM (minimal risk). However, since blood is usually the only sample analyzed in parents in order to determine heritability, it is more than likely that we are missing parental mosaicism events. Actually, several cases of apparently unaffected parents with very low levels of somatic mosaicism have been identified in CdLS^54^, for which a 4% of germline mosaicism has been estimated^55^. Thus, it is worth noting that deep sequencing of DNA derived from buccal cells or fibroblasts would be a reliable way to investigate somatic mosaicism in patients and parents, and subsequently, to estimate recurrence risk. In this context, recurrence risk of future pregnancies could be split into four groups based on the type of pathogenic variants found in the probands and their parents: high (parental constitutive variant), moderate (parental gonadosomic and/or germline mosaic variant), low (germline DNM) and minimal (PZM in child).

In conclusion, the high prevalence of mosaicism in CdLS as well as the likely purifying selection against disease-causing variants in blood should be considered when molecular diagnosis of the proband and familial co-segregation studies are planned.

## Material and methods

### Patient recruitment and data collection

All mosaic patients were recruited as a part of an international collaboration between investigators from Spain, Germany and Italy. The study was performed according to the Declaration of Helsinki protocols and was approved by each Regional Ethics Committee of Clinical Research. Informed consent was obtained from parents or guardians of all individuals included in this study, and from all the parents in which inheritance of the variants have been evaluated. Patients with mosaic disease-causing variants in *NIPBL* were phenotyped either by a clinical geneticist, a pediatrician or a trained physician. Clinical data were collected using a standard restricted-term questionnaire, and detailed phenotypes of the individuals were entered by the patients’ clinician using the Human Phenotype Ontology (HPO) nomenclature. Clinical scores for CdLS were calculated according to the published international consensus guidelines^14^. For the prevalence study, we collected retrospective data from the database of our National Center for Cornelia de Lange syndrome. The study was approved by the Ethics Committee of Clinical Research from the Government of Aragón (CEICA; PI15/00707). In total, 43 patients were included in the study. The inclusion criteria were: (i) Patients with a clinical score for CdLS above 8. If not enough clinical data were available to calculate the clinical score and the pediatrician suspected of CdLS, patients for whom CdLS had been suggested as the most probable clinical diagnoses in the sorted suggestion list of Face2Gene (FDNA) (https://www.face2gene.com). A previous study has proven that a diagnosis of CdLS was within the top-1 predicted syndrome for 83.7% of the individuals molecularly confirmed as CdLS^56^. (ii) Patients with at least two different biological sources of DNA available (blood, saliva, buccal swabs and/or fibroblasts).

### Molecular diagnosis

The new mosaic patients described in this article were molecularly diagnosed in their respective centers. DNA source (peripheral blood, buccal swab, urine cells, fibroblasts or skeletal muscle) and genomic technique used (exome sequencing, custom panel sequencing or Sanger sequencing) are presented in Table 2.

For the retrospective study, all patients were subjected to molecular analysis in the Clinical Genetics and Functional Genomics Group in the University of Zaragoza.

### DNA isolation

Genomic DNA was isolated from blood lymphocytes using conventional phenol–chloroform isoamyl alcohol method, from oral mucosa epithelial cells using prepIT.L2P (DNA Genotek Inc.), and/or from fibroblasts samples using PureLink™ Genomic DNA kit (Invitrogen) according to the manufacture’s protocols. Quality and concentration of gDNA were determined using both, the Qubit Fluorometric Quantitation (Thermo Fisher Scientific) and Nanodrop 2000 (Thermo Fisher Scientific).

### Next-generation sequencing

A panel of gene amplicons specific for CdLS was designed through the AmpliSeq™ Designer online tool (https://ampliseq.com/login/login.action). Designed panel was spanning 249.25 kb of the selected genomic sequencing including: *NIPBL* (NM_133433.3), *SMC1A* (NM_006306.3), *SMC3* (NM_058243.2), *RAD21* (NM_006265.2), *HDAC8* (NM_018486.2), *BRD4* (NM_058243.2), *ANKRD11* (NM_001256183.1) and *MAU2* (NM_015329.3). Library preparation, emulsion PCR, bead enrichment, and chip loading were automatically performed on an Ion Chef™ instrument (Thermo Fisher Scientific) using Ion AmpliSeq™ Kit for Chef DL8 and 530™ Kits (Thermo Fisher Scientific) according to the manufacture’s protocols. Templates were sequenced on an Ion S5™ XL sequencer (Thermo Fisher Scientific) using 530™ Kits with read length set at 200 and eight samples per chip. Sequencing results were analyzed using Ion Torrent Suite™, Ion Reporter™ and IGV (Broad Institue)^57^ softwares. Human Genome Variation Society (HGVS) (www.hgvs.org) nomenclature guidelines were used to name the genetic changes at the DNA level and the predicted resulting protein. The variants were classified according to the ACMG recommendations and detailed information provided in public databases gnomAD (https://gnomad.broadinstitute.org/), OMIM (https://omim.org/), ClinVar (https://www.ncbi.nlm.nih.gov/clinvar/), dbSNP (https://www.ncbi.nlm.nih.gov/snp/), LOVD (https://www.lovd.nl/), and relevant scientific literature. The in silico analyses were performed using online tools: Polyphen-2 (http://genetics.bwh.harvard.edu/pph2/), SIFT (https://sift.bii.a-star.edu.sg/), MutationTaster (http://www.mutationtaster.org/), PROVEAN Tool (http://provean.jcvi.org/index.php) and VarSome (https://varsome.com/).

### Sanger sequencing

Independent PCR followed by Sanger sequencing was performed to confirm those reportable SNVs and indel variants detected by NGS and for co-segregation analyses. Primers were designed using the Primer-Blast in silico tool (https://www.ncbi.nlm.nih.gov/tools/primer-blast/) and checked in the UCSC In-Silico PCR tool (https://genome.ucsc.edu/cgi-bin/hgPcr). All primer sequences and annealing temperatures are presented in Supplementary Table 2. PCR products were sequenced on ABI3730xl Capillary Electrophoresis Sequencing System (Applied Biosystems) according to manufacturer´s protocol. Sequences were analyzed and compared with the reference sequences using the Analysis Module Variant Analysis (VA) software (Applied Biosystem) and Ensembl and NCBI databases.

### MLPA and CGH array

If the panel did not detect causal variants, multiplex ligation-dependent probe amplification (MLPA) and/or comparative genomic hybridization array (aCGH) were done. MLPA was used to search for genomic copy number variations in *NIPBL* gene. The SALSA P141/P142 NIPBL MLPA kit (MRC-Holland, Amsterdam, The Netherlands) was used following the manufacturer’s instructions, the reaction products were separated by capillary electrophoresis on Abi Prism 3130XL Analyzer (Applied Biosystems) and the results obtained were analysed using GeneMapper software (Thermo Fisher Scientific). aCGH analyses were performed with the qChip Post oligonucleotide microarray (Quantitative Genomic Medicine Laboratories, Barcelona, Spain).

### Systematic review

We systematically searched the literature in the databases PubMed, Web of Sciences and EMBASE from 2005 to 2020. The search strategy included the key words of “de Lange Syndrome”, “mosaicism”, “somatic mosaicism”, and “postzygotic mutation”. We also manually checked the reference lists from relevant articles and reviews. Trials, case reports, cohort studies and reviews were included. After full-text review, papers containing patients diagnosed with CdLS according to standard clinical criteria and carrying pathogenic variants in one of the major causative genes of CdLS were included. Asymptomatic familial cases and germline mosaicisms were excluded.

### ClinVar variants analysis

For the statistical analyses, we referred to *NIPBL* disease-associated variants deposited in ClinVar. We employed the available database ClinVar VCF_2.0 file (version: 20210412) to obtain *NIPBL* variants (n = 835). We next selected the pathogenic and likely pathogenic *NIPBL* variants (n = 378), and filtered out variants according to allele origin: de novo (24) and germline (326); and molecular consequence: frameshift (n = 116), missense (n = 59), nonsense (n = 82) and splice site (n = 42). Altogether, our study considers 299 *NIPBL* variants described in ClinVar.

### Statistical analyses and figures

Statistical analyses and graphics were produced with GraphPad Prism 6 software. Data sets were compared by chi-square test when corresponded. Differences were considered statistically significant at *p* values below 0.05. ∗*p* < 0.05; ∗∗*p* < 0.01; ∗∗∗*p* < 0.001; ∗∗∗∗*p* < 0.0001. All statistical analyses are explained in the figure legends. Figure 1e was generated using the Pymol Molecular Graphics System (https://pymol.org/; Schrödinger, LLC, Portland, OR) and the information contained in the Protein Data Bank structure 6WGE^38^.

## Supplementary Information

Supplementary Tables.
