# Supplementary material for: Clinical relevance of postzygotic mosaicism in Cornelia de Lange syndrome and purifying selection of NIPBL variants in blood
Source: Sci Rep. 2021 Jul 29;11:15459. doi: 10.1038/s41598-021-94958-z (PMC8322329; doi:10.1038/s41598-021-94958-z)
Supplement: Supplementary file 1 — Supplementary Tables. [file 41598_2021_94958_MOESM1_ESM.pdf]

SUPPLEMENTARY INFORMATION

Clinical relevance of postzygotic mosaicism in Cornelia de Lange syndrome and purifying selection of *NIPBL* variants in blood

Ana Latorre-Pellicer[1], Marta Gil-Salvador[1], Ilaria Parenti[2], Cristina Lucia-Campos[1], Laura Trujillano[3], Iñigo Marcos-Alcalde[4,5], María Arnedo[1], Ángela Ascaso[3], Ariadna Ayerza-Casas[6], Rebeca Antoñanzas-Pérez[1], Cristina Gervasini[7], Maria Piccione[8], Milena Mariani[9], Axel Weber[10], Deniz Kanber[2], Alma Kuechler[2], Martin Munteanu[2], Katharina Khuller[2], Gloria Bueno-Lozano[3], Beatriz Puisac[1], Paulino Gómez-Puertas[4], Angelo Selicorni[9], Frank J. Kaiser[2,11], Feliciano J. Ramos[3]*, Juan Pié[1]*

Supplementary Table 1: Systematic literature review of postzygotic mosaicism in CdLS.

Sex/Age	Gene	Nucleotide change	Protein change	Type	E/I	Method	Blood	Buccal Cells	Fibroblasts	Urine	Muscle	Phenotype	Novel or Reported	Ref
M/12 months	*NIPBL*	Deletion exon 2 to 32		deletion	2 to 32	MLPA/FISH/aCGH	Mut	n.d	n.d	n.d	n.d	Classic	Novel	(25)
F/6	*NIPBL*	c.19del	p.(His7Metfs*19)	frameshift	2	Sanger	WT	Mut	n.d	n.d	n.d	na	Novel	(27)
M/12	*NIPBL*	c.42delG	p.(Ile16Leufs*8)	frameshift	2	NGS	n.d	22%	n.d	n.d	n.d	Classic	Novel	#I5
M/19	*NIPBL*	c.133C>T	p.(Arg45*)	nonsense	3	NGS	n.d	19%	n.d	n.d	n.d	Classic	Reported	#I7
M/14	*NIPBL*	c.358+1G>A	p.(Ile77Metfs*5)	splice variant	I4	Sanger	WT	Mut	n.d	n.d	n.d	na	Reported	(27)
na/na	*NIPBL*	c.358+3G>T		splice variant	I4	Sanger	WT	Mut	n.d	n.d	n.d	na	Reported	(12)
na/na	*NIPBL*	c.459-9G>A		splice variant	I5-6	Sanger	WT	Mut	n.d	n.d	n.d	na	Novel	(12)
na/na	*NIPBL*	c.742_745dup	p.(His249Profs*9)	frameshift	7	Sanger	WT	Mut	n.d	n.d	n.d	na	Novel	(12)
na/na	*NIPBL*	c.790del	p.(Met264*)	nonsense	8	Sanger	WT	Mut	n.d	n.d	n.d	na	Novel	(12)
M/49	*NIPBL*	c.816_817delGA	p.(Arg273Ilefs*12)	frameshift	8	NGS	n.d	31%	n.d	n.d	n.d	Classic	Novel	#I6
F/41	*NIPBL*	c.869-2A>G	p.(Gly290_Lys498del)	splice variant	I8	NGS	23%	51%	n.d	n.d	n.d	Mild	Reported	(26, 54)
na/na	*NIPBL*	c.1345C>T	p.(Gln449*)	nonsense	9	Sanger	WT	Mut	n.d	n.d	n.d	na	Novel	(12)
M/na	*NIPBL*	c.1435C>T	p.(Arg479*)	nonsense	9	Sanger	WT	n.d	n.d	n.d	n.d	na	Reported	(13)
						Pyro	19%	n.d	n.d	n.d	n.d			
						NGS	15%	n.d	n.d	n.d	n.d			
M/26	*NIPBL*	c.2294G>A	p.(Arg765Lys)	missense	10	NGS	26%	n.d	n.d	n.d	n.d	Non-classic	Novel	#I1
na/na	*NIPBL*	c.2389C>T	p.(Arg797*)	nonsense	10	Sanger	WT	Mut	n.d	n.d	n.d	na	Reported	(12)
M/5	*NIPBL*	c.2435_2436insA	p.(Ser813Valfs*5)	frameshift	10	Sanger	WT	n.d	Mut	n.d	n.d	Classic	Novel	#I11
						NGS	<2%	n.d	35.99%	n.d	n.d			
F/17	*NIPBL*	c.2602C>T	p.(Arg868*)	nonsense	10	NGS	n.d	23%	n.d	n.d	n.d	Classic	Reported	#I8
M/1	*NIPBL*	c.2827delA	p.(Ser943Valfs*11)	frameshift	10	Sanger	Mut	Mut	n.d	n.d	n.d	Classic	Novel	(11)
						Pyro	10%	33%	n.d	n.d	n.d			
M/5	*NIPBL*	c.3021delA	p.(Lys1007Asnfs*37)	frameshift	10	Sanger	WT	n.d	Mut	n.d	n.d	Classic	Novel	#I10
						NGS	2%	n.d	46.50%	n.d	n.d			

na/na	*NIPBL*	c.3327del	p.(Asp1110Metfs*63)	frameshift	11	Sanger	WT	Mut	n.d	n.d	n.d	na	Novel	(12)
M/18 months	*NIPBL*	c..4020_4024delinsAGTGTA	p.(Lys1341Valfs*13)	frameshift	17	Sanger	WT	Mut	n.d	n.d	n.d	Classic	Novel	(27)
F/2	*NIPBL*	c.4094T>A	p.(Leu1365*)	nonsense	18	Sanger	WT	n.d	Mut	n.d	n.d	Classic	Novel	(37)
						NGS	n.d	17%	n.d	n.d	n.d			
M/32	*NIPBL*	c.4399A>T	p.(Lys1467*)	nonsense	20	Sanger	WT	Mut	n.d	Mut	n.d	Classic	Novel	(36)
						NGS	n.d	30%	n.d	n.d	n.d			
na/na	*NIPBL*	c.4543G>T	p.(Glu1515*)	nonsense	21	Sanger	WT	Mut	n.d	n.d	n.d	na	Reported	(12)
F/19	*NIPBL*	c.4751T>G	p.(Leu1584Arg)	missense	23	Sanger	WT	WT	Mut	n.d	n.d	Classic	Novel	(37)
						NGS	n.d	13%	n.d	n.d	n.d			
F/6	*NIPBL*	c.5328+1G>C	p.(Met1743Serfs*17)	splice variant	I27	Sanger	WT	WT	Mut	n.d	n.d	Mild	Novel	(37)
						NGS	n.d	21%	n.d	n.d	n.d			
						SNaPshot	n.d	n.d	n.d	Mut	n.d			
F/na	*NIPBL*	c.5440C>T	p.(Arg1814*)	nonsense	29	NGS	WT	20%	n.d	n.d	n.d	Classic	Reported	(26)
F/na	*NIPBL*	c.5483G>A	p.(Arg1828Gln)	missense	29	Sanger	WT	Mut	WT	n.d	n.d	Classic	Reported	(36)
						Pyro	2%	20.40%	16%	n.d	n.d			
						NGS	n.d	n.d	15%	n.d	n.d			
F/na	*NIPBL*	c.6206T>A	p.(Ile2069Asn)	missense	35	NGS	n.d	21%	n.d	n.d	n.d	Classic	Novel	(26)
F/3	*NIPBL*	c.6635T>A	p.(Val2212Glu)	missense	39	NGS	2%	29%	n.d	n.d	n.d	Classic	Reported	#I2
F/5	*NIPBL*	c.6647A>C	p.(Tyr2216Ser)	missense	39	Sanger	WT	n.d	n.d	n.d	n.d	Classic	Novel	(24)
						Pyro	11%	23%	47%	n.d	n.d			
						NGS	23%	n.d	n.d	n.d	n.d			
M/na	*NIPBL*	c.6893G>A	p.(Arg2298His)	missense	40	NGS	15%	38%	n.d	n.d	n.d	na	Reported	(13)
M/18	*NIPBL*	c.7168G>A	p.(Ala2390Thr)	missense	42	Sanger	WT	n.d	Mut	n.d	Mut	Classic	Reported	#I9
						NGS	<2%	n.d	23%	n.d	37.90%			
na/na	*NIPBL*	c.7168G>A	p.(Ala2390Thr)	missense	42	Sanger	WT	Mut	n.d	n.d	n.d	na	Reported	(12)
na/na	*NIPBL*	c.7263+5G>A		splice variant	I42	Sanger	WT	Mut	n.d	n.d	n.d	na	Novel	(12)
M/52	*NIPBL*	c.7328_7329insA	p.(Glu2444Glyfs*19)	frameshift	43	NGS	n.d	n.d	16.17%	n.d	n.d	Classic	Novel	#I3

M/4.02	*NIPBL*	c.7373_7374del	p.(Ser2458Cysfs*4)	frameshift	43	NGS	WT	12%	n.d	n.d	n.d	na	Novel	(13, 40)
M/4	*NIPBL*	c.7621delC	p.(Gln2541Argfs*9)	frameshift	44	NGS	< 2%	26%	n.d	n.d	n.d	Classic	Novel	#I4
M/6	*SMC1A*	c.793_795delGAG	p.(Glu265del)	nonframeshift	7	NGS	4%	60%	n.d	n.d	n.d	Classic	Novel	(42)
M/18.3	*SMC1A*	c.1585_1587del	p.(Lys529del)	nonframeshift	11	NGS	n.d	10%	n.d	n.d	n.d	na	Novel	(13, 40)
F/na	*SMC3*	c.703_705del	p.(Thr235del)	nonframeshift	9	NGS	n.d	n.d	n.d	n.d	n.d	na	Novel	(13)
F/4	*ANKRD11*	c.5483G>T	p.(Ser1828*)	nonsense	9	Pyro	32%	n.d	46%	n.d	n.d	Classic	Novel	(41)
						NGS	31%	n.d	n.d	n.d	n.d			
M/na	*KMT2A*	c.4012+1G>A		splice variant	I7	NGS	WT	48%	n.d	n.d	n.d	Mild	Novel	(26)

Studies and mosaicism genetic variants included in this study. Biological sample analyzed, diagnosis method used, allele frequency and CdLS phenotype for each individual are indicated. Since Sanger sequencing is not a quantitative method, only detection (Mut) or not detection (WT) of the causative variant is indicated. NIPBL RefSeq NM_133433; SMC1A RefSeq NM_006306; SMC3 RefSeq NM_005445; ANKRD11 RefSeq NM_001256183; KMT2A RefSeq NM_001197104. Abbreviations: M, male; F, female; na, not available; Sanger: Sanger sequencing; Pyro: pyrosequencing; NGS: Next-Generation Sequencing; WT: wild-type or variant not detected; Mut: pathogenic variant detected; n.d., not determined.

Supplementary Table 2: List of primers sequences used in Sanger sequencing

Individual #	Exon/Intron	Forward (5'-3')	Revers (5'-3')	Annealing (°C)
NIPBL				
CdLS 1	35	TAACTGGACCTTTACGTGCAA	GCTCACACAATGTTGCACTAC	55
CdLS 2	30	TTCTAGTCTTGTGTCCAGGGC	ATCAACATTTAGGTGCAATAA	55
CdLS 3	10	AGGTGAGAGCCGCCCTGAAACTC	CACGAGGACTGTCAGGTCTTGA	58
CdLS 4	9	GTGAAACCACCACAACTG	TGAGCAGCATTTAGTGGGC	55
CdLS 5	36	TGGCATGACTGTAAGCACTCA	AGAGGACCACGGTGGATAATC	60
CdLS 6	32i	GTTCTGTAACGTTGGTAAATGGT	GGTTCTTTTAAATCATACAGTCCA	58
CdLS 7	29	GTCTGAGGTTGTTGCTGTAGA	ATGATATTGCAAGGGCTATTC	55
CdLS 8	40	CAGATTAAGAACCATTGAGCC	GCAGTAATCATAACCCAAGAG	55
CdLS 9	47	GGCTTCAGTGTTCAGTGGATG	TTTGCCCAACATTTCCTTC	58
CdLS 10	36	TGGCATGACTGTAAGCACTCA	AGAGGACCACGGTGGATAATC	60
CdLS 11	9	CAGGACAGACTTCAAAAACACC	CCAAATCTCATATAGTTGTTTCAG	55
CdLS 12	39	GAAGTACCTGCTCTAATGC	GAATATGCTAAAGCCCAGC	55
CdLS 13	40	CAGATTAAGAACCATTGAGCC	GCAGTAATCATAACCCAAGAG	55
CdLS 14	21i	TTGGCAAACACAGTATCGTG	GATCGCGCCACTGCACTC	55
CdLS 15	20i	TTGGCAAACACAGTATCGTG	GATCGCGCCACTGCACTC	55
CdLS 16	45	TCCAAATACGTTGTTTCCATAG	TCAATGTGAAGGAGATAGTTAT	52
CdLS 17	27i	ACGAAAGGCTCCAAAGTATG	ACTGCTGCTTCTCGGACAC	55
CdLS 18	27i	ACGAAAGGCTCCAAAGTATG	ACTGCTGCTTCTCGGACAC	55
CdLS 19	46	CCACACCAAACTACTGCCATAG	CATTTTACGTAATACGCTGCG	60
CdLS 20	29	GTCTGAGGTTGTTGCTGTAGA	ATGATATTGCAAGGGCTATTC	55
CdLS 21	15	ATTCAGGGTTTACTTGAGGTT	AGTCCATGCCTCTTTCAATGCAG	60
CdLS 22	29	GTCTGAGGTTGTTGCTGTAGA	ATGATATTGCAAGGGCTATTC	55
CdLS 23	42	ATGAAGCTAGCCTCAGAATGT	CAAAATTTCCCCTTCACTTCTGA	55
CdLS 24	39	GAAGTACCTGCTCTAATGC	GAATATGCTAAAGCCCAGC	55
CdLS 25	10	TGAATCAGGGGACTCAAGGG	AGGGAACTTCTTGATTTGTCCTC	58
CdLS 26	10	AGGTGAGAGCCGCCCTGAAACTC	CACGAGGACTGTCAGGTCTTGA	58
SMC1A				
CdLS 27	4	AATGGGTAAGGTGAACTGGG	AAACAGCACGGCCTCTTGGT	58
CdLS 28	5	AATGGGTAAGGTGAACTGGG	AAACAGCACGGCCTCTTGGT	58
CdLS 29	15	CCAATGCAGTCAAGGTAGCT	GATGTCAAGCTAGAGGCTCA	58
CdLS 30	13	CAGGCTCAGTACTGGAGATT	AACCTAGGCCAGGAATGTGT	58
SMC3				
CdLS 31	22	GAGCACAAGTACTAGAGAGG	CTAGGTGGCAGAATTCCAAC	57
HDAC8				
CdLS 32	5	CAAGGGTTAGATCCTTGGC	CTACCAGTTGCCTAGAAGC	52
CdLS 33	4	TAGGTTAGGTTGTTGTCAC	GATTTCTTCAGAATGCCTT	55
ANKRD11				
CdLS 34	9	CCTGTCGGAGAAGAAGAGCC	CTCCTCTTTGTACAGTCTCAG	54
RAD21				
CdLS 35	11	CCTACATTGTACTGAGGGTAG	GGACTGTCTATATCCTGTTGC	58
